# Chlorophyll fluorescence-based control of greenhouse supplemental lighting improves energy use efficiency in lettuce

**DOI:** 10.3389/fpls.2026.1854406

**Published:** 2026-07-13

**Authors:** Suyun Nam, Rhuanito Soranz Ferrarezi

**Affiliations:** Department of Horticulture, University of Georgia, Athens, GA, United States

**Keywords:** biofeedback system, chlorophyll fluorescence, controlled environment agriculture, dynamic lighting, greenhouse lighting, light energy use efficiency, plant-driven greenhouse control

## Abstract

Plant-driven lighting control has been proposed as a strategy to regulate supplemental light-emitting diode (LED) intensity according to real-time plant physiological status. This study developed a multiple linear regression (MLR) model to predict quantum yield of photosystem II (Φ_PSII_) from environmental variables and evaluated its integration into a chlorophyll fluorescence-based biofeedback light control. The model incorporated light intensity, CO_2_ concentration, air temperature, vapor pressure deficit, short-term light history, and diurnal effects. In a greenhouse validation experiment, supplemental lighting was regulated using either direct chlorophyll fluorometer measurements of Φ_PSII_ (sensor-based control) or Φ_PSII_ values predicted by the machine learning model (ML-based control), and compared with a constant photosynthetic photon flux density (PPFD) treatment. Both sensor- and ML-based control stabilized photochemical activity across the photoperiod relative to constant PPFD. Although plant growth did not differ among treatments, sensor-based ETR control achieved the highest energy use efficiency for LED lighting in this study. These findings demonstrate the feasibility of integrating predictive ML models into plant-based lighting control systems and indicate that sensor-based biofeedback control improved the energy-use efficiency of greenhouse supplemental lighting without compromising crop growth.

## 1 Introduction

Controlled environment agriculture (CEA) allows consistent, high-yield, and year-round crop production by precisely regulating environmental conditions such as light, temperature, humidity, and CO_2_ concentration (Neo et al., 2022). Artificial lighting is one of the critical components in CEA system, particularly in greenhouses where photosynthetic lighting is often required when natural sunlight is limited due to seasonal or weather variation. However, in greenhouse production, electricity use for supplemental lighting can account for up to 45-85% of total energy use, depending on the climate (Katzin et al., 2021).

In greenhouse systems, supplemental light intensity is commonly regulated to maintain a constant photosynthetic photon flux density (PPFD) using dimmable light emitting diode (LED) fixtures and quantum sensors (Pinho et al., 2013). This approach enables rapid adjustments in response to fluctuating solar radiation while achieving daily light integral (DLI) requirements. Although this method supports consistent crop growth, more dynamic light control strategies have emerged to enhance photosynthetic efficiency and reduce energy use. For instance, extending a photoperiod with lower PPFD levels or gradually increasing light intensity over time can maximize photosynthetic efficiency and light interception throughout the cultivation period (Jin et al., 2023; Weaver and van Iersel, 2020).

Recently, plant-driven climate control strategies have attracted attention for their ability to dynamically adjust resource delivery in response to real-time crop physiological demand (Kernbach, 2024; Sun et al., 2025). Chlorophyll fluorescence (CF) is a powerful tool for real-time assessment of photosynthetic performance and is sensitive to various abiotic stressors (Adireddy et al., 2025; Maxwell and Johnson, 2000). In addition, due to its non-invasive and rapid measurement method, CF parameters such as electron transport rate (ETR) and quantum yield of photosystem II (Φ_PSII_) have been utilized to monitor photochemical status and regulate LED light intensities; an approach referred to as CF-based biofeedback lighting control (van Iersel et al., 2016b). In previous studies, such biofeedback systems have dynamically adjusted LED light levels by capturing crop-specific photosynthetic responses, light acclimation patterns, and diurnal changes in photosynthetic efficiency (Nam et al., 2025). Another study reported that these systems can reduce excessive light application when plants are stressed, thereby minimizing photoinhibition and improving plant growth without unnecessary energy use (Stevens et al., 2026).

However, the biofeedback systems also have limitations for large-scale greenhouse applications. The pulse amplitude modulated (PAM) fluorometers used in previous studies measure CF only from a small portion of a single leaf, which may not represent the overall photosynthetic performance of the crop canopy (Nam et al., 2025). Since light regulation relies on this localized measurement, any leaf damage or atypical photosynthetic behavior can lead to either excessive or insufficient light delivery to the entire growing area. In addition, previous studies recommended measuring CF parameters at 15-minute intervals (Nam et al., 2025; van Iersel et al., 2016b). Shorter intervals can induce photoinhibition because each CF measurement involves high-intensity saturating light pulses (van Iersel et al., 2016a). For example, when CF was measured every 2 minutes, the biofeedback system progressively reduced LED light intensity and even turned the lights off due to excessive stress on the leaf. A 15-minute interval may be appropriate for indoor systems, but it may not be sufficiently responsive for greenhouse conditions where environmental factors change rapidly (Nam et al., 2025). Moreover, PAM fluorometers are relatively expensive, and multiple units would be required to capture spatial variability within commercial-scale greenhouses, which may limit their economic feasibility for large-scale implementation.

Machine learning (ML) offers a promising approach to predicting plant physiological responses from greenhouse environmental variables and imaging data. For example, Amir et al. (2021) demonstrated that sap flow in cherry tomatoes could be predicted using ML models based on environmental and irrigation variables such as solar radiation, temperature, humidity, irrigation water electrical conductivity (EC), and drainage volume. Such predictive models could potentially enable growers to adjust irrigation strategies dynamically, thereby improving water-use efficiency. Likewise, ML models have been developed to predict photosynthetic performance. For example, CO_2_ assimilation rate and the maximum quantum efficiency of photosystem II (*F*_v_/*F*_m_) have been predicted using various inputs, including climate variables, physiological responses, and multispectral imaging data (Pappert et al., 2025; Wu et al., 2023; Yang et al., 2022). These models are typically proposed for rapid, noninvasive monitoring of photosynthetic and abiotic stress status without direct and leaf-level measurements, allowing broader spatial monitoring. However, most studies have focused on model development and performance comparison, whereas the application of ML models in real-time greenhouse control remains limited.

Recent initiatives, such as the “Autonomous Greenhouse Challenge” led by Wageningen University & Research, have further demonstrated the potential of artificial intelligence (AI) in greenhouse management. AI algorithms were developed to control key operations, including lighting, HVAC, and irrigation, without human intervention and have been shown to outperform experienced growers in net profit by improving resource use efficiency and crop quality (Hemming et al., 2019; Petropoulou et al., 2023). Among these factors, optimizing LED lighting was the most significant contributor to increased production and net profit (Hemming et al., 2020). This highlights the critical role of data-driven supplemental lighting regulation in greenhouse crop production.

The objectives of this study were (1) to develop an ML model to predict Φ_PSII_ based on greenhouse climate and temporal variables that can be readily measured, and (2) to integrate the model into the biofeedback light control system by using the predicted CF values instead of direct measurements. We hypothesized that ML-based control would achieve target CF parameters with accuracy comparable to direct measurements, while also improving plant growth and/or energy use efficiency compared with constant PPFD control.

## 2 Materials and methods

### 2.1 Plant materials and growth conditions

The experiment was conducted at the University of Georgia (College of Agricultural and Environmental Sciences, Department of Horticulture, Controlled Environment Agriculture Crop Physiology and Production Laboratory) located in Athens, Georgia, USA (33 93’11.36” N, 83 36’39.28” W). Lettuce (*Lactuca sativa* ‘Cherokee’) seeds (Johnny’s Selected Seeds, Waterville, ME, USA) were sown weekly into 96-cell trays containing a peat-perlite substrate (Fafard 1P; SunGro Horticulture, Agawam, MA, USA). The seedlings were then grown in a walk-in growth chamber under white LED light bars (RAY series with Physiospec indoor spectrum; Fluence Bioengineering, Austin, TX, USA) with a spectral distribution of 39% red, 40% green, 18% blue, and 3% far-red radiation. Under a 16-h photoperiod, canopy-level PPFD was maintained at 320 μmol·m^-2^·s^-1^, corresponding to a DLI of 18.4 mol·m^-2^·d^-1^. Plants were irrigated daily using an automated ebb-and-flow subirrigation system supplying a 15N-2.2P-12.4K fertilizer (Jack’s Professional^®^ LX 15-5–15 Cal-Mg LX; JR Peters, Allentown, PA, USA) at 100 mg·L^-1^ N (nitrogen). During seedling cultivation, the growth chamber environment averaged 24.2 ± 0.3 °C for air temperature (Temp), 1.24 ± 0.06 kPa for vapor pressure deficit (VPD), and 831.6 ± 12.5 μmol·mol^-1^ for carbon dioxide concentration (CO_2_) (mean ± standard deviation [SD]).

After three weeks, seedlings were transplanted into 10-cm square containers using the same soilless substrate and moved to a glass-covered greenhouse in Athens, Georgia, USA (33°57′26.676″ N, 83°22′36.48″ W). Following one week of acclimation period in the greenhouse, the 4-week-old plants were used for two types of experiment: 1) data collection for the development of ML models predicting Φ_PSII_, and 2) a validation experiment comparing biofeedback systems based on ML-predicted and directly measured CF values, with an additional constant PPFD control treatment. To ensure consistent plant age across experiments, this entire process for preparing plant materials was repeated weekly. Each week, a new batch of plants was used for the data collection or the validation experiment. During both the model development and validation experiments, all greenhouse plants were irrigated daily using the same subirrigation system with a fertilizer concentration of 200 mg·L^-1^ N.

### 2.2 Data collection

Over a period of six consecutive weeks, four lettuce plants were placed in an ebb-and-flow subirrigation tray (125 × 125 × 10 cm, W × D × H) and used per week for data collection. A multi-environment sensor (SM-600; Apogee Instruments, Logan, UT, USA) was installed at the center of the tray to monitor Temp, VPD, and CO_2_ every 30 seconds, and 15-min averaged values were recorded. To regulate incoming sunlight intensity and maintain a DLI level appropriate for lettuce (≈ 16 mol·m^-2^·d^-1^), a 50% shade curtain was installed above the tray. Light levels were measured every 5 seconds using two pairs of quantum sensors: photosynthetically active radiation (PAR, 400–700 nm) and extended PAR (ePAR, 400–750 nm) sensors (SQ-500-SS and SQ-610-SS; Apogee Instruments, Logan, UT, USA). One set of each quantum sensor type was positioned directly next to the uppermost fully expanded leaf of each plant. Instantaneous light intensities measured at 15-minute intervals were designated PPFDi and ePPFDi, whereas PPFD15 and ePPFD15 denoted the average values over the preceding 15 minutes. Sub-daily accumulated light integrals, DLI_cum and eDLI_cum, were calculated at the same intervals to represent cumulative light received from dawn to the time of measurement.

CF was measured every 15 minutes using a multi-site pulse-amplitude modulated (PAM) fluorometer (MONITORING-PAM; Heinz Walz, Effeltrich, Germany). The uppermost fully expanded leaf of each plant was measured at the middle portion of the leaf lamina between secondary veins using four measuring heads, and data acquisition was automated using proprietary software (WinControl-3; Heinz Walz, Effeltrich, Germany).

**Figure 1:**
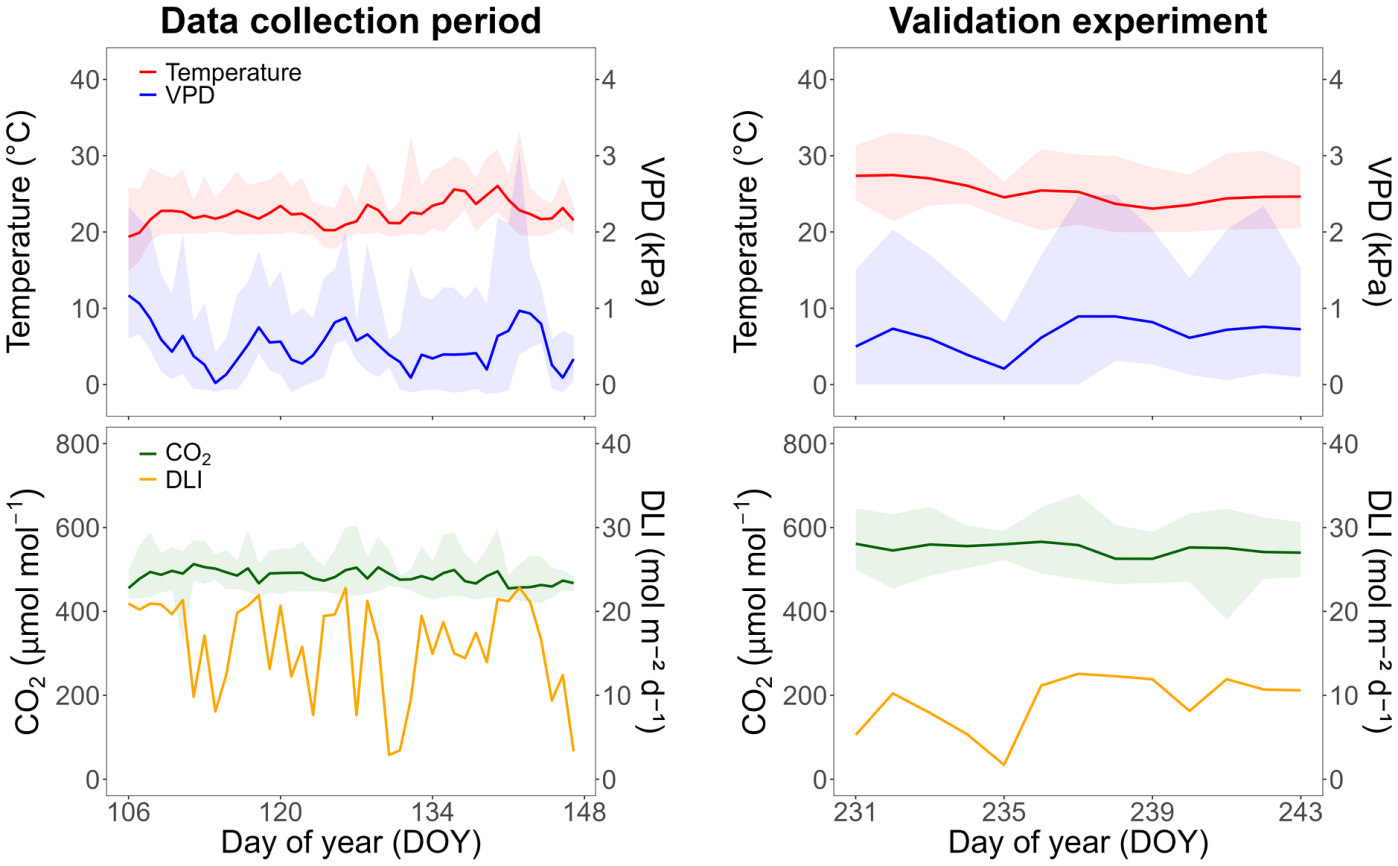
Environmental conditions during the data collection (left) and validation experiment (right). Air temperature, vapor pressure deficit (VPD), and carbon dioxide (CO_2_) concentration were recorded every 15 minutes. The data is summarized as daily means (solid lines) and daily minimum and maximum values (shaded areas).

All environmental sensors were connected to a datalogger (CR1000X; Campbell Scientific, Logan, UT, USA) for continuous data collection. Daily average environmental conditions and DLI during data collection and validation experiment are presented in **Figure 1**. All environmental variables and CF data were recorded simultaneously at 15-minute intervals (:00,:15,:30, and:45) throughout the 13.5 h photoperiod (06:45–20:15). Environmental variables, measured at the center of the tray, were shared among the four plants at each time point. A total of 5,079 data points from 24 lettuce plants were used to develop the model.

### 2.3 ML model development

A supervised ML approach using multiple linear regression (MLR) was developed to predict Φ_PSII_ based on environmental and temporal variables. Time-of-day variable was included as a binary categorical variable with two levels, “beforenoon” and “afternoon”, created by splitting the data at 13:30, corresponding to the approximate solar midday at the experimental site during the study period. Initial variable screening was performed by examining the bivariate relationships between Φ_PSII_ and each continuous predictor (**Table 1**). Stepwise regression based on Akaike Information Criterion (AIC) was performed using the stepAIC function in the MASS package in R (v 4.5.0; R Foundation for Statistical Computing, Vienna, Austria). Three selection approaches, forward selection, backward elimination, and bidirectional stepwise selection, consistently indicated the exclusion of PPFDi. To further address multicollinearity, models were manually refined, and the final variable set was selected based on AIC and variance inflation factor (VIF < 5).

**Table 1:** Coefficient of determination (R^2^) from simple linear regression models evaluating the bivariate relationship between each environmental predictor and quantum yield of photosystem II (Φ_PSII_).

Predictors	R^2^
ePPFD15	0.525
ePPFDi	0.488
PPFD15	0.417
PPFDi	0.406
CO_2_	0.163
Temp	0.0812
VPD	0.0435
eDLI_cum	0.0348
DLI_cum	0.0184

Each model was fitted independently using the full dataset without any data splitting or cross-validation.

Where: ePPFD15, 15-min average extended photosynthetic photon flux density (400–750 nm); ePPFDi, instantaneous ePPFD; PPFD15, 15-min average PPFD (400–700 nm); PPFDi, instantaneous PPFD; CO_2_, carbon dioxide concentration; Temp, air temperature; VPD, vapor pressure deficit; eDLI_cum, extended daily light integral accumulated from dawn to time of measurement (400–750 nm); and DLI_cum, daily light integral accumulated from dawn to time of measurement (400–700 nm).

**Figure 2:**
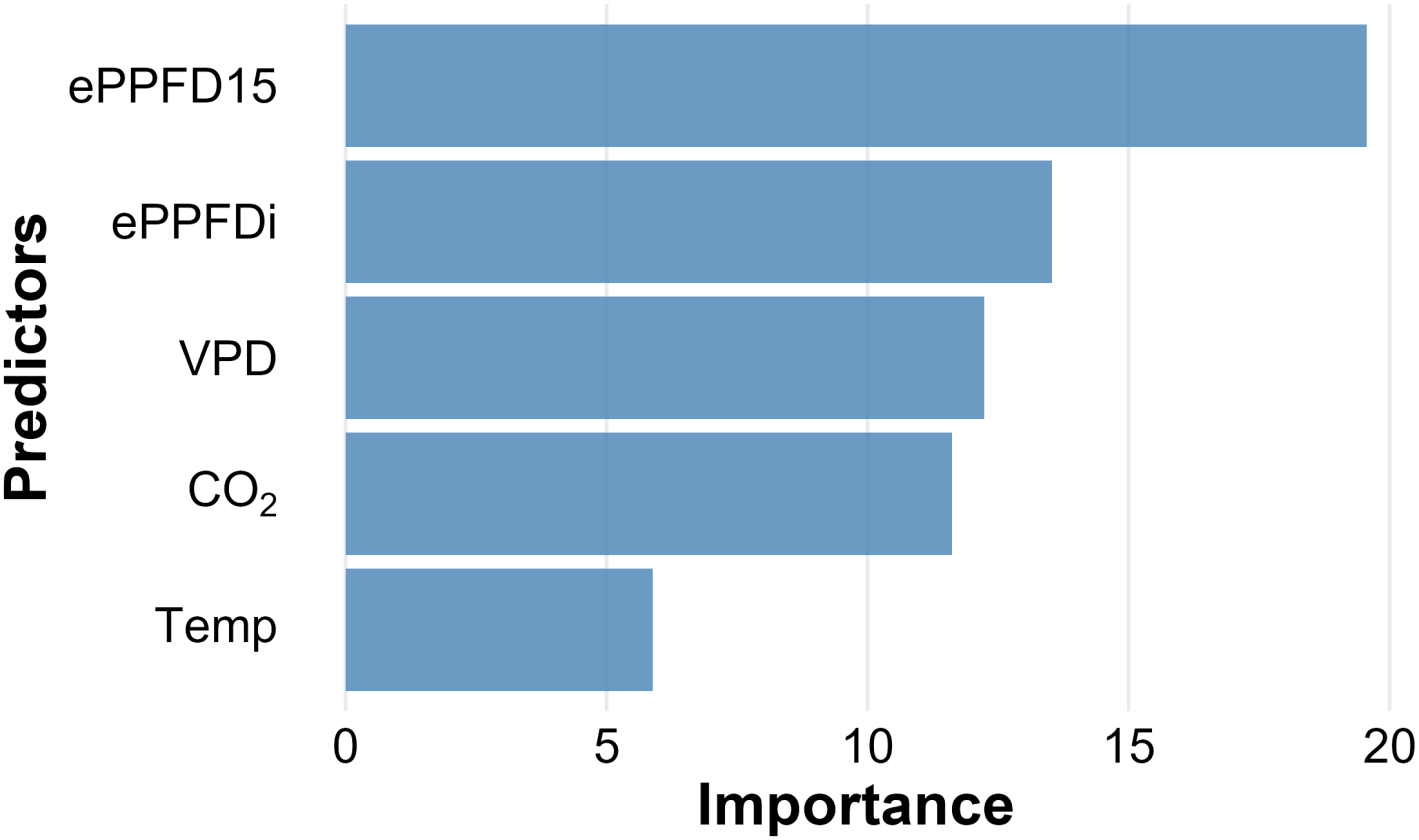
Variable importance for predicting the quantum yield of photosystem II (Φ_PSII_) from the final multiple linear regression (MLR) model. Importance values are based on standardized regression coefficients. Predictors include extended photosynthetic photon flux density averaged over 15 minutes (ePPFD15), instantaneous ePPFD (ePPFDi), vapor pressure deficit (VPD), carbon dioxide concentration (CO_2_), and air temperature (Temp). Higher values indicate stronger influence on the model.

The final MLR model included the following predictors: ePPFDi, ePPFD15, Temp, VPD, CO_2_, and Time-of-day. Variables were not normalized to preserve original units for direct use of regression coefficients in the validation experiment. The dataset was randomly split into training (70%) and testing (30%) sets. Five-fold cross-validation was conducted on the training data using the tidymodels package in R to assess model performance and stability. Hyperparameter tuning was not performed, and model performance was summarized by averaging evaluation metrics across folds. The model performance was evaluated using the root mean square error (RMSE), coefficient of determination (R^2^), and mean absolute error (MAE) on both the training and test datasets. Regression coefficients, along with their standard errors and *P*-values, are presented in **Table 2**. Variable importance was assessed using the vip package in R to identify the relative contribution of each predictor variable in the final model (**Figure 2**). Linearity and homoscedasticity were assessed using residuals-versus-fitted plots, while residual normality was evaluated using normal Q-Q plots. Multicollinearity in the final model was evaluated using VIF, and temporal autocorrelation was assessed using autocorrelation function (ACF) plots and the Durbin-Watson test.

**Table 2:** Regression coefficients for the multiple linear regression model used to predict the quantum yield of photosystem II (Φ_PSII_).

Predictors	Coefficients	Standard errors	*P*-values
Intercept	5.30 × 10^-1^	3.26 × 10^-2^	< 0.001
ePPFD15	-1.62 × 10^-4^	8.25 × 10^-6^	< 0.001
ePPFDi	-9.45 × 10^-5^	6.97 × 10^-6^	< 0.001
Temp	-3.80 × 10^-3^	7.66 × 10^-4^	< 0.001
VPD	5.03 × 10^-2^	4.08 × 10^-3^	< 0.001
CO_2_	5.71 × 10^-4^	5.82 × 10^-5^	< 0.001
Time-of-day^1^	5.80 × 10^-3^	3.72 × 10^-3^	0.119

Predictors included 15-min averaged and instantaneous extended photosynthetic photon flux density (ePPFD15 and ePPFDi), air temperature (Temp), vapor pressure deficit (VPD), carbon dioxide concentration (CO_2_), and Time-of-day.

¹Time-of-day was included as a binary categorical variable coded as “afternoon” (reference) and “beforenoon” (estimate shown). The positive coefficient indicates that Φ_PSII_ tended to be higher in the beforenoon compared to the afternoon.

### 2.4 Validation experiment settings

A validation experiment was conducted in the same greenhouse to evaluate the applicability of the ML model for real-time supplemental lighting control. The experiment was conducted on a 9 m × 1.5 m bench divided into five blocks. Each block contained five experimental units (0.36 m × 1.5 m) separated by vertical aluminum panels to prevent light interference between treatments. Supplemental light treatments were randomized within each block. A 60% shade net was installed above the bench to achieve the DLI within an appropriate range by limiting excess solar radiation. Environmental conditions were measured using the same multi-environment sensor (SM-600; Apogee Instruments, Logan, UT, USA) positioned above the third block, between the supplemental LED lights and the shade net. Plants were irrigated daily using an automated ebb-and-flow system with the same nutrient solution as during the data-collection period.

White LED light bars (RAY series with Physiospec Greenhouse spectrum; Fluence Bioengineering, Austin, TX, USA) were installed above each experimental unit. Each bar was 1.1 m in length and had a spectral composition of 37% red, 39% green, 21% blue, and 3% far-red. Five quantum sensors (SQ-500-SS; Apogee Instruments, Logan, UT, USA) were positioned 25 cm below the LED bars, beside the plant canopy, and at the same height as the CF measurement spots in each treatment of the third block. These sensors measured the combined PPFD from sunlight and supplemental LED lighting every 15 seconds. All quantum and environmental sensors were connected to one of two dataloggers (CR1000X; Campbell Scientific, Logan, UT, USA) for Φ_PSII_ prediction and lighting control.

### 2.5 CF measurement

CF parameters were measured using two types of the PAM fluorometers (Heinz Walz, Effeltrich, Germany) for the validation experiment. Two MINI-PAM units were connected to the datalogger via RS-232 interface for real-time measurement of CF parameters used in the sensor-based lighting control. All measurements were conducted every 15 minutes during the 13-hour photoperiod and were automated using proprietary software either LoggerNet (Campbell Scientific) or WinControl-3 (Heinz Walz). Measurements were taken on the uppermost fully expanded leaves of each plant at the middle portion of the leaf, as described above. The CF parameters included Φ_PSII_ and ETR, which were calculated using the following equations (Hendrickson et al., 2004):


$$\begin{array}{l} \Phi_{\text{PSII}}=\left(F_{\text{m}}^{\prime }-F_{\text{s}}\right)/F_{\text{m}}^{\prime }\\ \Phi_{\text{NO}}=F_{\text{s}}/F_{\text{m}}\\ \Phi_{\text{NPQ}}=F_{\text{s}}/F_{\text{m}}^{\prime }-F_{\text{s}}/F_{\text{m}} \end{array}$$

where *F*_s_ is the steady-state fluorescence yield under actinic light, $F_{\text{m}}^{\prime }$ is the maximum fluorescence of a light-adapted leaf, and *F*_m_ is the maximum fluorescence of a dark-adapted leaf.

### 2.6 Sensor-based lighting control

Two sensor-based biofeedback logic modules were implemented in the datalogger to control supplemental light intensity based on either ETR or Φ_PSII_. Real-time CF measurements were obtained every 15 minutes during the photoperiod, and the datalogger computed the new target PPFD (PPFD_t_) accordingly. For the ETR-based control, PPFD_t_ was calculated using the ratio between the target ETR (ETR_t_) and current ETR (ETR_c_) values as follows:


$$\begin{array}{l} \text{If }{\text{ETR}}_{\text{c}}>{\text{ETR}}_{\text{t}}\times 1.05\text{ or }{\text{ETR}}_{\text{c}}<{\text{ETR}}_{\text{t}}\times 0.95\text{ then}\\ {\text{PPFD}}_{\text{t}}={\text{PPFD}}_{\text{c}}\times \left({\text{ETR}}_{\text{t}}/{\text{ETR}}_{\text{c}}\right) \end{array}$$

This approach assumes a linear relationship between ETR and PPFD under sub-saturating light levels. The conditional clause minimized unnecessary light adjustments when ETR_c_ was within ±5% of the target.

For the Φ_PSII_-based control, PPFD_t_ was computed from the deviation between the target (Φ_PSII, t_) and current Φ_PSII_ (Φ_PSII, c_) values, using the slope (*k*) derived from the empirical relationship between PPFD and Φ_PSII_ based on the dataset collected for ML model development, as follows:


$$\begin{array}{l} \text{If }{\text{Φ}}_{\text{PSII,c}}>{\text{Φ}}_{\text{PSII,t}}\times 1.01\text{ or }{\text{Φ}}_{\text{PSII,c}}<{\text{Φ}}_{\text{PSII,t}}\times 0.99\text{ then}\\ {\text{PPFD}}_{\text{t}}={\text{PPFD}}_{\text{c}}+\left({\text{Φ}}_{\text{PSII,t}}-{\text{Φ}}_{\text{PSII,c}}\right)/k \end{array}$$

The slope *k* was calculated from the linear regression of Φ_PSII_ against PPFD (Φ_PSII_ = 0.7347 − 0.0002827 × PPFD), giving *k* = 0.0002827. This allowed conversion of a difference in Φ_PSII_ to an equivalent PPFD adjustment. Likewise, ± 1% threshold around Φ_PSII, t_ was applied to avoid over-reactive adjustments in PPFD.

### 2.7 ML-based lighting control

The MLR model developed was implemented to predict the Φ_PSII_ in real time using environmental and temporal inputs. The model equation was applied as:


$$\text{Predicted }{\text{Φ}}_{\text{PSII}}=\text{Intercept}+{\text{β}}_{1}\text{ ePPFD}15+{\text{β}}_{2}\text{ ePPFDi}+{\text{β}}_{3}\text{ Temp}+{\text{β}}_{4}\text{ VPD}+{\text{β}}_{5}{\text{ CO}}_{2}+{\text{β}}_{6}\text{ Time-of-day}$$

where Time-of-day is a binary variable (1 for before 13:30, 0 otherwise).

All input variables were collected in real time. Temp, VPD, and CO_2_ were measured every minute, whereas ePPFD were measured every 15 seconds using ePAR sensors (SQ-610-SS; Apogee Instruments, Logan, UT, USA), installed only in the ML-based lighting treatments. ePPFD_15_ was calculated as the average ePPFDi over the preceding 15 min, based on measurements taken every 15 s. The predicted Φ_PSII_ and ETR were then used to calculate PPFDt, following the same control logic as for sensor-based lighting control, with all computations performed in real time by the datalogger.

### 2.8 Supplemental lighting control

Each datalogger was connected to an analog output module (SDM-AO4A, Campbell Scientific, Logan, UT, USA) to control the LED light levels. The module sent 0–10 V direct current dimming signals to the LED drivers, each of which powered five LED bars from the five blocks. Supplemental lighting was provided for 13 h per day (07:00–20:00). To dynamically adjust light intensity in response to changing sunlight, the dimming signal was updated every 15 seconds based on the following equation:


$$\text{New dimming signal}=\text{Old dimming signal}\times \left({\text{PPFD}}_{\text{t}}/{\text{PPFD}}_{\text{c}}\right)$$

where PPFD_t_ is the target PPFD calculated from the biofeedback system or constant PPFD treatment, and PPFD_c_ is the current light intensity measured by the quantum sensors.

At the beginning of the photoperiod, all treatments were adjusted to a total PPFD of 350 μmol·m^-2^·s^-1^ for 30 minutes to minimize transient fluctuations before stable operation of the biofeedback light control, as previously demonstrated (Nam et al., 2025). In sensor-based control, PPFD_t_ was adjusted every 15 min because saturating pulses during CF measurements can induce photoinhibition if applied too frequently. In contrast, the ML-based control predicted CF parameters using non-invasive environmental measurements, allowing PPFD_t_ to be adjusted every 15 seconds. Regardless of the PPFD_t_ adjustment interval, the dimming signal was recalculated every 15 seconds using PPFD_c_ to respond promptly to fluctuations in sunlight. In the constant PPFD treatment, PPFD_t_ was fixed at 350 μmol·m^-2^·s^-1^, and the dimming signal was directly adjusted every 15 s based on the ration of PPFD_t_ to PPFD_c_.

### 2.9 Harvest and energy use parameters

All plants were harvested 15 days after the supplemental lighting treatments. Shoot fresh weight was recorded, and shoot dry weight was determined after drying at 80 °C for 72 h. Leaf chlorophyll and anthocyanin contents were measured on three uppermost fully expanded leaves per plant using handheld meters (CCM-200 plus and ACM-200 plus; Opti-Sciences, Hudson, NH, USA), and the mean value was used for each experimental unit.

A linear relationship between dimming signals and power consumption was determined using a power meter (P4400; P3 International Corporation, New York, NY, USA). Power consumption was measured at dimming signals ranging from 0 to 10 V in 1 V increments, with five replicates at each level. The resulting equation, Power consumption (W) = 7.93 × dimming signal (V) (R^2^ = 0.99), was used to estimate instantaneous power use for the supplemental LED lighting, which was then integrated over time to calculate total electricity consumption per plant. Energy use efficiency (g·kWh^-1^) was calculated as the ratio of shoot fresh and dry weight to the amount of electricity consumed.

### 2.10 Experimental design and statistical analysis

Five supplemental lighting treatments were arranged in a randomized complete block design (RCBD) with five blocks (n = 5). The treatments consisted of two types of biofeedback regulation: a sensor-based control using real-time CF measurements and an ML-based predictive control. Each control strategy was implemented using either an ETR-based logic or a Φ_PSII_-based logic. Accordingly, the treatments included sensor-based biofeedback control targeting an ETR of 100 μmol·m^-2^·s^-1^ (BFB-ETR 100) and ML-based control targeting the same ETR (ML-ETR 100), as well as sensor-based control maintaining a target Φ_PSII_ of 0.68 (BFB-Φ_PSII_ 0.68) and ML-based control targeting the same Φ_PSII_ value (ML-Φ_PSII_ 0.68). In addition, a constant PPFD treatment was included, where supplemental lighting was adjusted in response to changing sunlight to maintain a target PPFD of 350 μmol·m^-2^·s^-1^ (PPFD 350), representing a conventional light control strategy for comparison. Specific target ETR, Φ_PSII_, and PPFD values were selected prior to the experiment, based on preliminary tests, to provide DLI levels sufficient for lettuce growth and achieve comparable DLIs across treatments.

All statistical analyses were performed using R (v4.5.0; R Foundation for Statistical Computing, Vienna, Austria). Linear mixed-effects models were applied, treating treatment as a fixed effect and block as a random effect. Harvest and energy use parameters were analyzed using one-way analysis of variance (ANOVA), and pairwise comparisons were performed with Tukey’s Honestly Significant Difference (HSD) test at a 95% confidence level.

## 3 Results

### 3.1 Dataset characteristics and model development

**Figure 3:**
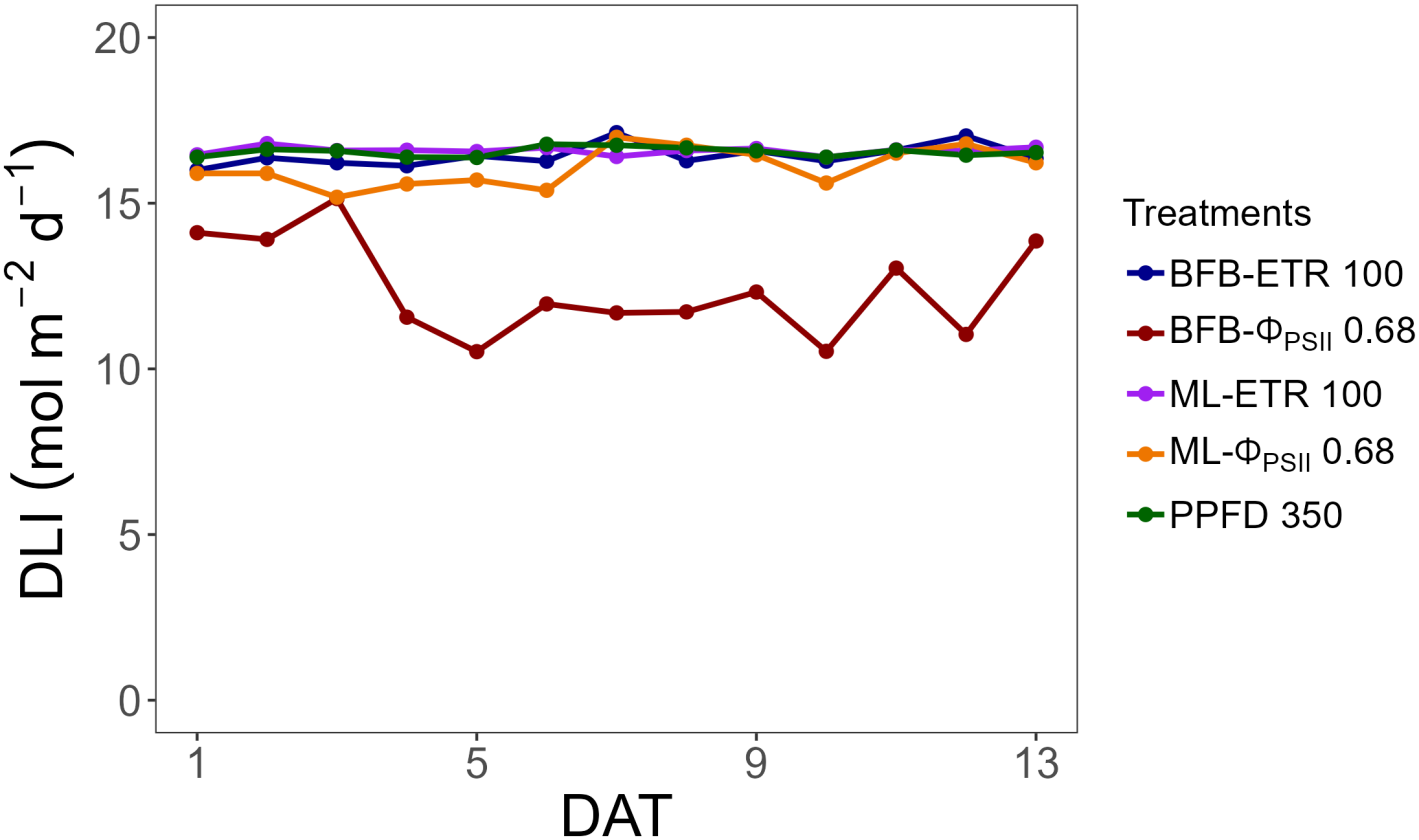
Daily light integral (DLI) during 13 days after treatment (DAT) under five lighting treatments. BFB-ETR 100 and ML-ETR 100 indicate biofeedback control targeting an electron transport rate (ETR) of 100 μmol·m^-2^·s^-1^ using chlorophyll fluorometer-based and machine learning-based regulation, respectively. BFB-Φ_PSII_ 0.68 and ML-Φ_PSII_ 0.68 indicate the same control strategies targeting a quantum yield of photosystem II (Φ_PSII_) of 0.68. PPFD 350 represents a constant light control strategy maintaining a photosynthetic photon flux density (PPFD) of 350 μmol·m^-2^·s^-1^.

Environmental conditions varied widely during the data collection period, providing a broad range for training the ML model (**Figure 1**). Temperature and CO_2_ showed relatively modest variability, averaging 22.4 ± 2.5 °C (range 14.8–33.2 °C) and 483 ± 38 μmol·mol^-1^ (range 325–605 μmol·mol^-1^), respectively. In contrast, VPD and DLI exhibited substantial day-to-day fluctuations. VPD averaged 0.51 ± 0.43 kPa (range 0–3.1 kPa), and DLI averaged 15.9 ± 5.7 mol·m^-2^·d^-1^ (range 2.9–22.8 mol·m^-2^·d^-1^). Environmental conditions during the validation experiment overlapped with those observed during the data collection period but exhibited differences in their distribution and ranges (**Supplementary Figure 1**). Temperature averaged 25.2 ± 3.0 °C (range 20.0–33.1 °C), and VPD averaged 0.65 ± 0.56 kPa (range 0–2.5 kPa) during the validation experiment (**Figure 1**). CO_2_ was moderately higher, averaging 549 ± 51 μmol·mol^-1^ (range 380–680 μmol·mol^-1^), but still overlapped with most values from the data collection period. DLI averaged 9.2 ± 3.3 mol·m^-2^·d^-1^ (range 1.7–12.6 mol·m^-2^·d^-1^) without supplemental lighting and 15.6 ± 1.8 mol·m^-2^·d^-1^ with supplemental lighting (**Figure 3**), closely matching the mean DLI during the data collection.

**Figure 4:**
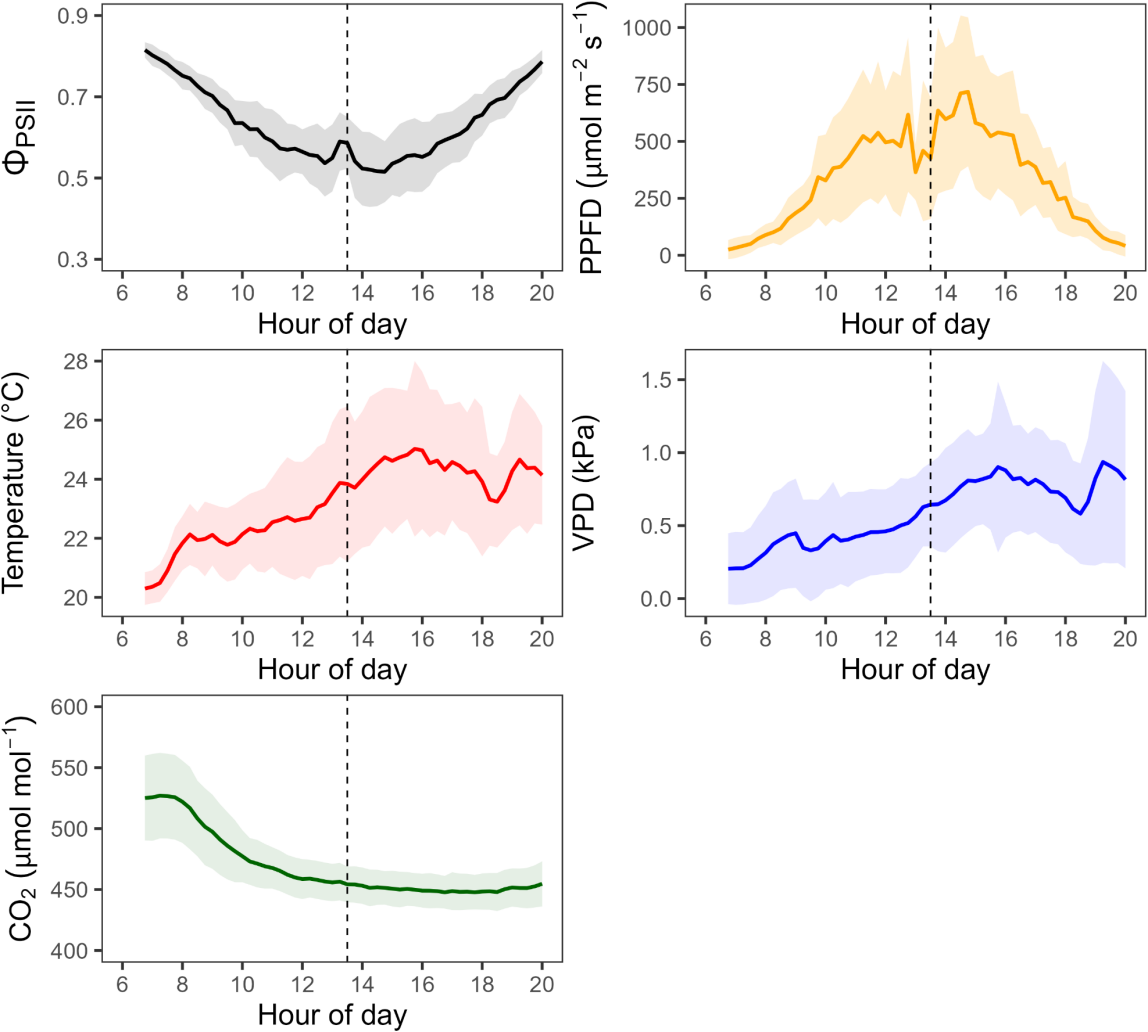
Diurnal patterns of quantum yield of photosystem II (Φ_PSII_) and environmental variables (photosynthetic photon flux density [PPFD], air temperature, vapor pressure deficit [VPD], and carbon dioxide [CO_2_] concentration) during the data collection period. Solid lines indicate the mean across days, and shaded areas represent ± 1 standard deviation (SD) among days (n = 42).

Diurnal patterns of environmental conditions were visualized by averaging values at each hour of day across the data collection period (**Figure 4**). PPFD exhibited a largely symmetric, near-sinusoidal pattern, with a midpoint at approximately 13:30, corresponding to solar noon at the experimental location. A localized reduction in PPFD was observed around 13:00, likely due to transient shading from the greenhouse structure over the measurement area. Temp and VPD increased progressively during the photoperiod, whereas CO_2_ declined. Consistent with these asymmetric diurnal patterns in environmental variables, except for light intensities, Φ_PSII_ displayed a modest post-noon decline. The average Φ_PSII_ was 0.658 before 13:30 and 0.624 after 13:30.

**Figure 5:**
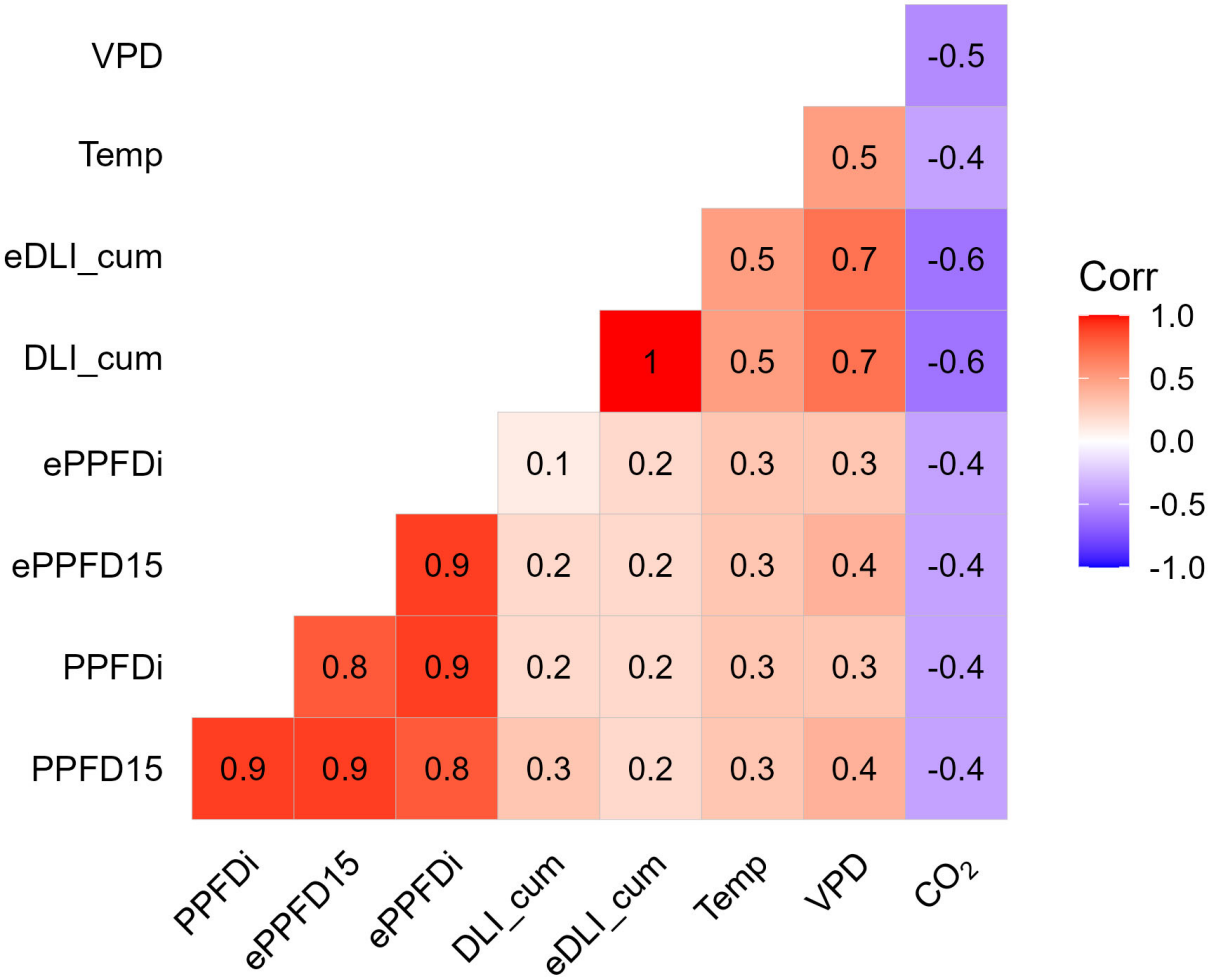
Pearson correlation matrix showing pairwise relationships among light- and environment-related variables used for model development. Blue and red colors indicate positive and negative correlations, respectively. PPFDi and PPFD15 denote instantaneous and 15-min averaged photosynthetic photon flux density, respectively. DLI_cum represents cumulative daily light integral. The prefix “e” indicates extended wavebands including far-red radiation. Temp, VPD, and CO_2_ indicate air temperature, vapor pressure deficit and carbon dioxide concentration, respectively.

Correlation analysis revealed strong positive relationships among light intensity-related variables (ePPFDi, ePPFD15, PPFDi, and PPFD15) and among cumulative light variables (eDLI_cum and DLI_cum) (**Figure 5**). Temp and VPD were also positively correlated with each other and negatively correlated with CO_2_, reflecting their opposing diurnal trends (**Figure 4**). Since cumulative light variables increased over time, DLI_cum and eDLI_cum showed positive relationships with Temp and VPD and a negative relationship with CO_2_ (**Figure 5**).

Bivariate relationships between individual environmental predictors and Φ_PSII_ were analyzed independently to provide an initial assessment of relative influence prior to multivariate modeling (**Table 1**). Light intensity-related variables exhibited the highest R^2^ values, followed by CO_2_, Temp, and VPD, whereas cumulated light variables showed the lowest R^2^. Across both ePPFD- and PPFD-based metrics, previous 15-min averaged light intensity variables (ePPFD15 and PPFD15) consistently explained more variation in Φ_PSII_ than instantaneous measurements (ePPFDi and PPFDi). In addition, light intensity metrics that included far-red radiation (ePPFD15 and ePPFDi) were associated with higher R^2^ values than their PPFD-based variables (PPFD15 and PPFDi).

### 3.2 Model performance

Overall, the final MLR model integrated short-term light environments, atmospheric conditions, and a temporal predictor to predict Φ_PSII_. The selected predictors included instantaneous and 15-min-averaged ePPFD (ePPFDi and ePPFD15), along with Temp, VPD, and CO_2_. A binary Time-of-day variable was retained in the final model to account for the diurnal asymmetry of Φ_PSII_. In contrast, PPFD- and DLI-based variables were not retained in the final model.

**Figure 6:**
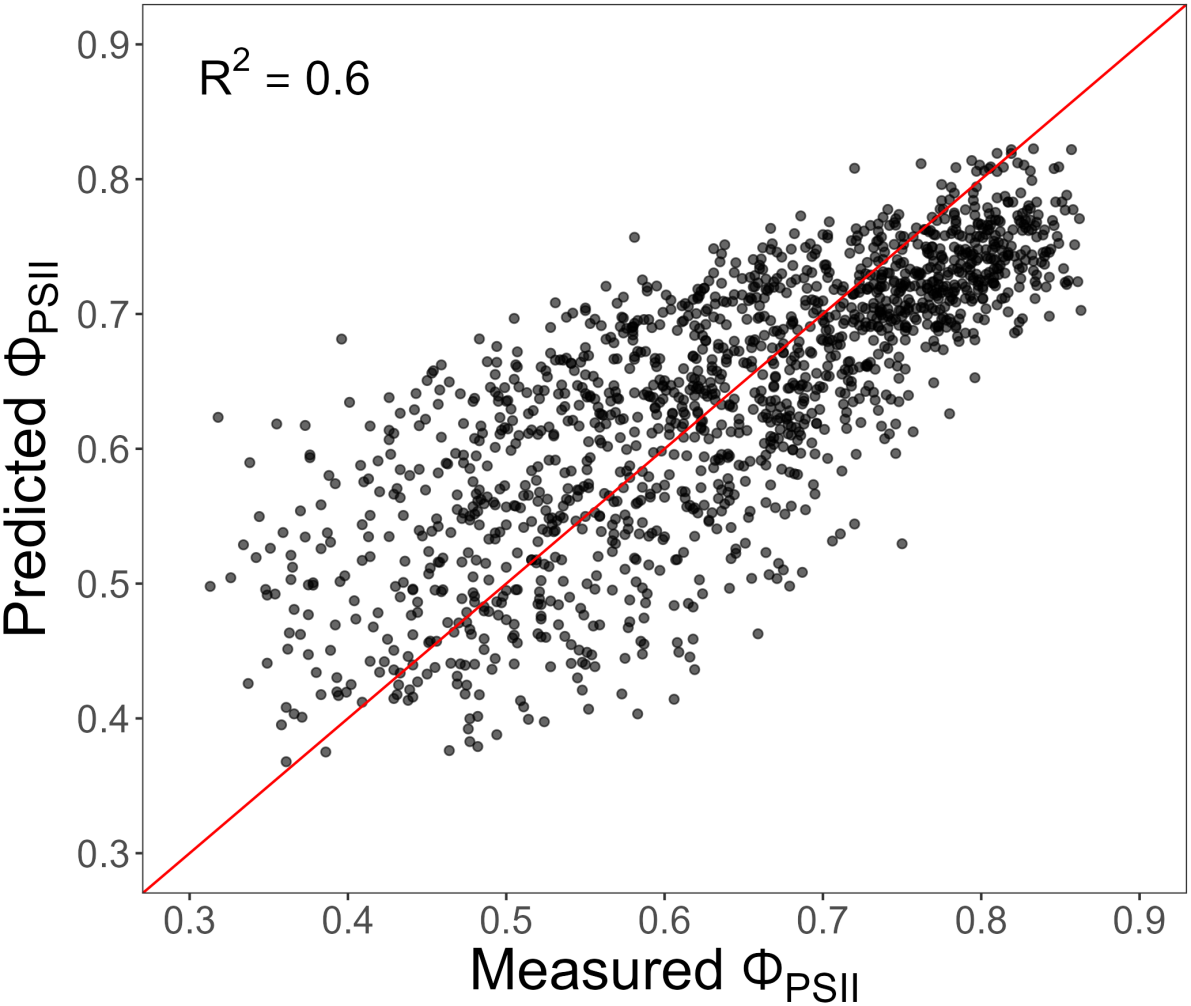
Relationship between measured and predicted quantum yield of photosystem II (Φ_PSII_) from the multiple linear regression (MLR) model. Each point represents a single observation from the testing dataset, and the red 1:1 line indicates perfect agreement.

Model performance was evaluated using a training-testing data split (**Table 3**). The model exhibited comparable predictive performance between the training and testing datasets, with RMSE values of 0.083 and 0.080 and R^2^ values of 0.56 and 0.60, respectively. Predicted Φ_PSII_ values followed measured values, with observations distributed around 1:1 line and no strong systematic bias across the prediction range (**Figure 6**). Diagnostic evaluation indicated no substantial violations of model assumptions, including linearity, residual normality, and multicollinearity (**Supplementary Figures 2, 3**; **Supplementary Table 1**). Temporal autocorrelation was not detected based on the Durbin-Watson test (DW = 1.999, *P* = 0.491).Variable importance analysis indicated that light intensity-related predictors contributed most strongly to model predictions (**Figure 2**). Among these, ePPFD15 had the highest importance, followed by ePPFDi. VPD and CO_2_ showed intermediate importance, whereas Temp had the lowest importance among the continuous predictors.

**Table 3:** Performance metrics of the final multiple linear regression model predicting the quantum yield of photosystem II (Φ_PSII_).

Datasets	RMSE	R^2^	MAE
Training	0.083	0.56	0.067
Testing	0.080	0.60	0.065

Values represent root mean square error (RMSE), coefficient of determination (R^2^), and mean absolute error (MAE) for both training and testing datasets.

Regression coefficients revealed negative associations between Φ_PSII_ and ePPFD15, ePPFDi, and Temp, whereas positive coefficients were observed for VPD and CO_2_ (**Table 2**). The positive coefficient of the Time-of-day indicates that Φ_PSII_ tended to be higher during the beforenoon compared to the afternoon.

### 3.3 Supplemental light control performance

Both sensor-based and ML-based control strategies maintained the target ETR of 100 μmol·m^-2^·s^-1^ throughout the experimental period, with average ETR values of 100.9 ± 11.1 (measured) for BFB-ETR 100 and 101.6 ± 8.7 μmol·m^-2^·s^-1^ (predicted) for ML-ETR 100 (**Table 4**).

**Table 4:** Chlorophyll fluorescence parameters and lighting conditions averaged over a 13-day period.

Treatments	ETR/Φ_PSII_ source	ETR (µmol·m^-2^·s^-1^)	Φ_PSII_	PPFD (µmol·m^-2^·s^-1^)	DLI (mol·m^-2^·day^-1^)
BFB-ETR 100	Measured	100.9 ± 11.1	0.687 ± 0.032	351.1 ± 19.4	16.4 ± 0.3
BFB-Φ_PSII_ 0.68	Measured	72.9 ± 23.6	0.646 ± 0.039	265.3 ± 72.1	12.4 ± 1.5
ML-ETR 100	Predicted	101.6 ± 8.7	0.681 ± 0.026	354.3 ± 18.7	16.1 ± 1.8
ML-Φ_PSII_ 0.68	Predicted	97.7 ± 21.0	0.678 ± 0.018	343.4 ± 61.5	15.5 ± 1.9
PPFD 350	Predicted	101.0 ± 9.8	0.677 ± 0.029	353.5 ± 13.9	16.0 ± 1.8

Parameters include electron transport rate (ETR), quantum yield of photosystem II (Φ_PSII_), photosynthetic photon flux density (PPFD), and daily light integral (DLI).

ETR and Φ_PSII_ were directly measured for the sensor-based treatments (BFB-ETR 100 and BFB- Φ_PSII_ 0.68) using fluorometers, whereas values for the other treatments were predicted using the machine-learning model. Values are means ± standard deviations. DLI values were calculated from daily means (n = 13), whereas all other parameters were derived from 15-min interval measurements (n = 676), representing temporal variability within each treatment.

For the Φ_PSII_-based strategies, the ML-Φ_PSII_ 0.68 achieved an average predicted Φ_PSII_ of 0.678 ± 0.018, closely matching the target value (**Table 4**). In contrast, BFB-Φ_PSII_ 0.68 resulted in a lower measured Φ_PSII_ of 0.646 ± 0.039 and was associated with lower average PPFD and DLI over the experimental period (**Figure 3**). In all other treatments, average PPFD and DLI were comparable at approximately 350 μmol·m^-2^·s^-1^ and 16 mol·m^-2^·day^-1^, respectively.

**Figure 7:**
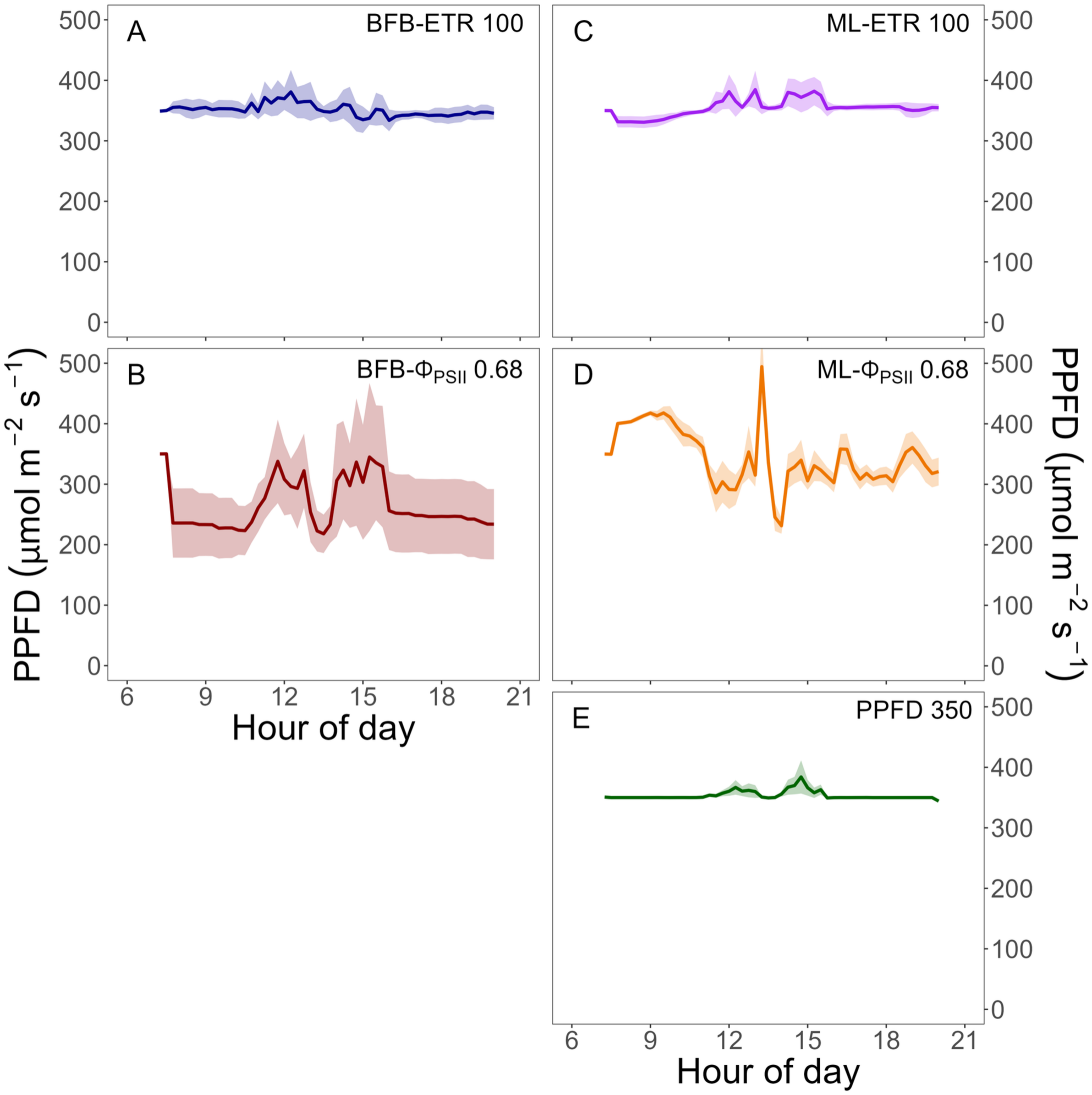
Diurnal patterns of photosynthetic photon flux density (PPFD) under five lighting control strategies, summarized across 13 days. Data were aggregated across days at each time of day (15-min resolution), with solid lines representing the mean PPFD and shaded areas indicating ±1 standard deviation (SD). BFB-ETR 100 and ML-ETR 100 indicate biofeedback control targeting an electron transport rate (ETR) of 100 μmol·m^-2^·s^-1^ using chlorophyll fluorometer-based and machine learning-based regulation, respectively. BFB-Φ_PSII_ 0.68 and ML-Φ_PSII_ 0.68 indicate the same control strategies targeting a quantum yield of photosystem II (Φ_PSII_) of 0.68. PPFD 350 represents a constant light control strategy maintaining a PPFD of 350 μmol·m^-2^·s^-1^.

**Figure 8:**
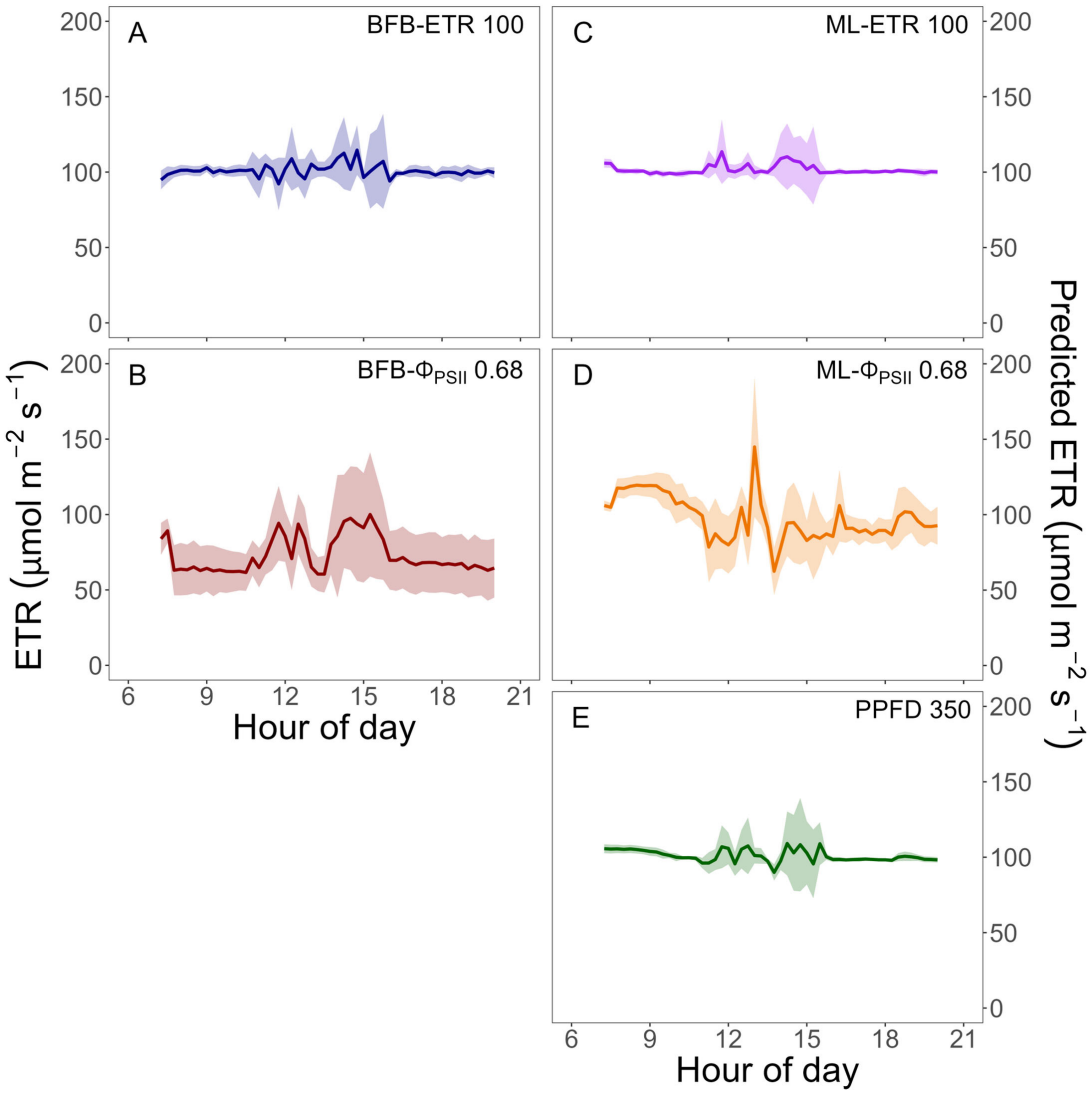
Diurnal patterns of electron transport rate (ETR) under five lighting control strategies, summarized across 13 days. Data were aggregated across days at each time of day (15-min resolution), with solid lines representing the mean ETR and shaded areas indicating ±1 standard deviation (SD). ETR was measured directly using chlorophyll fluorometers in panels A–B and predicted using the machine learning model in panels C–E. BFB-ETR 100 and ML-ETR 100 indicate biofeedback control targeting an ETR of 100 μmol·m^-2^·s^-1^ using chlorophyll fluorometer-based and machine learning-based regulation, respectively. BFB-Φ_PSII_ 0.68 and ML-Φ_PSII_ 0.68 indicate the same control strategies targeting a quantum yield of photosystem II (Φ_PSII_) of 0.68. PPFD 350 represents a constant light control strategy maintaining a photosynthetic photon flux density (PPFD) of 350 μmol·m^-2^·s^-1^.

**Figure 9:**
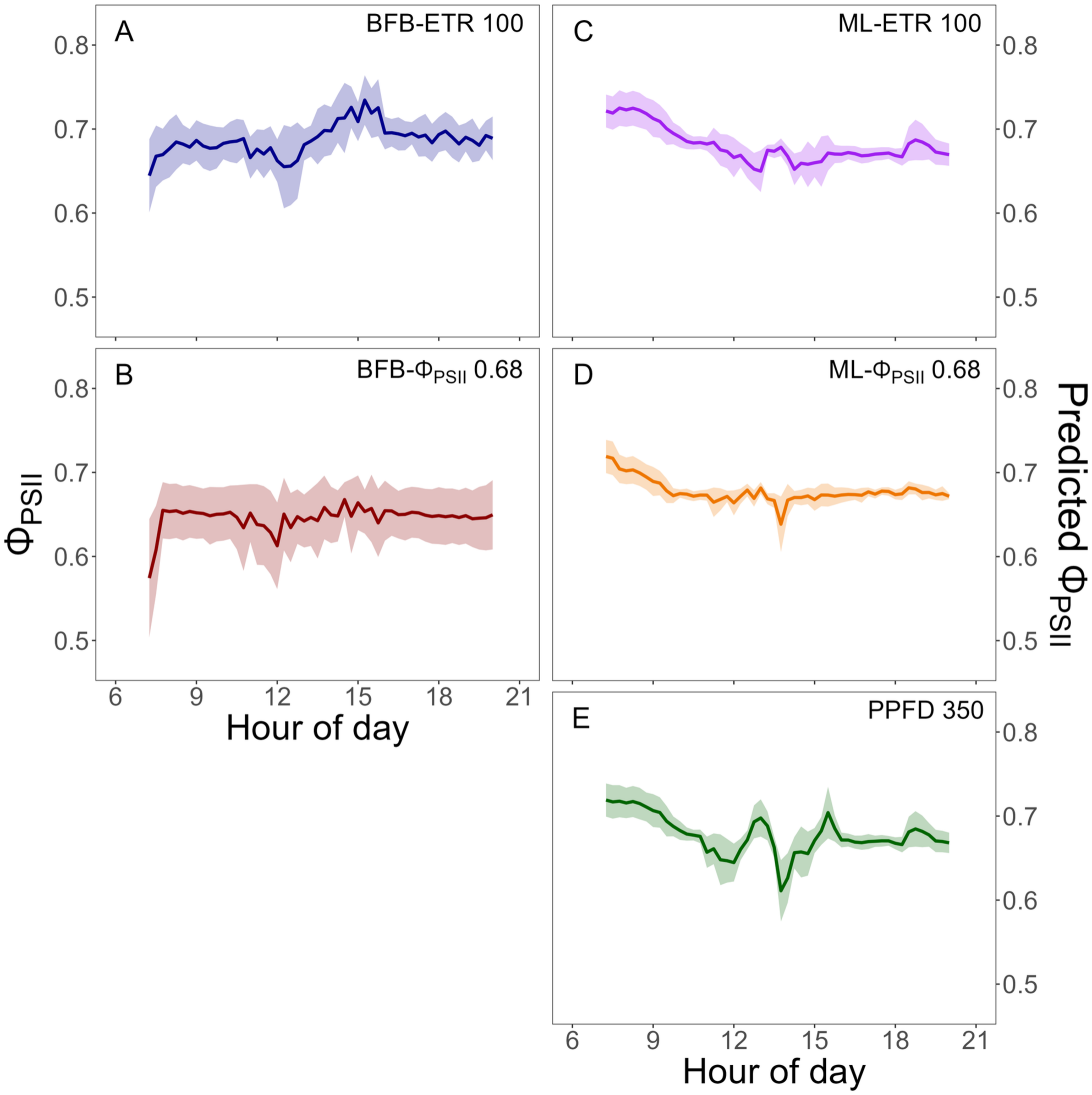
Diurnal patterns of quantum yield of photosystem II (Φ_PSII_) under five lighting control strategies, summarized across 13 days. Data were aggregated across days at each time of day (15-min resolution), with solid lines representing the mean Φ_PSII_ and shaded areas indicating ±1 standard deviation (SD). Φ_PSII_ was measured directly using chlorophyll fluorometers in panels A–B and predicted using the machine learning model in panels C–E. BFB-ETR 100 and ML-ETR 100 indicate biofeedback control targeting an electron transport rate (ETR) of 100 μmol·m^-2^·s^-1^ using chlorophyll fluorometer-based and machine learning-based regulation, respectively. BFB-Φ_PSII_ 0.68 and ML-Φ_PSII_ 0.68 indicate the same control strategies targeting a Φ_PSII_ of 0.68. PPFD 350 represents a constant light control strategy maintaining a photosynthetic photon flux density (PPFD) of 350 μmol·m^-2^·s^-1^.

**Figure 7** illustrates diurnal patterns of supplemental light adjustment within the photoperiod. The PPFD 350 treatment maintained a stable PPFD close to 350 μmol·m^-2^·s^-1^ throughout the photoperiod, with brief deviations during midday solar peaks (**Figure 7E**). In contrast, both BFB-ETR 100 and ML-ETR 100 exhibited slightly greater within-day variability in PPFD relative to constant PPFD treatment (**Figures 7A, C**), while ETR values were maintained near the target level of 100 μmol·m^-2^·s^-1^ (**Figures 8A, C**). In the ML-ETR 100 treatment, a transient increase in PPFD during the morning hours coincided with a pronounced decline in predicted Φ_PSII_ (**Figures 7C**, **9C**). By comparison, BFB-ETR 100 treatment showed an increase in measured Φ_PSII_ during the mid-photoperiod, followed by stabilization later in the day (**Figure 9A**).

Both BFB- and ML-Φ_PSII_ 0.68 treatments exhibited greater within-day fluctuations in supplemental light regulation compared with the ETR-based strategies and the constant PPFD treatment (**Figures 7B, D**). These fluctuations were also reflected in corresponding diurnal patterns of ETR (**Figures 8B, D**). Despite the dynamic light adjustments, measured and predicted Φ_PSII_ values in the Φ_PSII_-based treatments remained relatively stable throughout the photoperiod (**Figures 9B, D**). However, under the PPFD 350 treatment, predicted Φ_PSII_ exhibited pronounced diurnal variation throughout the photoperiod, compared with four biofeedback-based strategies (**Figure 9E**).

### 3.4 Lettuce growth and energy use efficiency

Shoot fresh weight, shoot dry weight, leaf chlorophyll content, and leaf anthocyanin content did not differ significantly among treatments (**Table 5**). Total supplemental light energy use varied among treatments, with ML-Φ_PSII_ 0.68 exhibiting the highest cumulative energy consumption, followed by PPFD 350 and ML-ETR 100. In contrast, sensor-based biofeedback treatments showed lower total energy use. Energy use efficiency, expressed as biomass production per unit of supplemental light energy, differed significantly among treatments. BFB-ETR 100 showed the highest energy use efficiency based on both fresh and dry weight, whereas PPFD 350 had the lowest values. Based on fresh weight, BFB-Φ_PSII_ 0.68 produced lower energy use efficiency than BFB-ETR 100, but the remaining treatments showed intermediate values with no significant differences.

**Table 5:** Growth and energy parameters measured 15 days after light treatments.

Target ETR	Shoot fresh weight (g)	Shoot dry weight (g)	Leaf chlorophyll content	Leaf anthocyanin content	Total energy use (kWh)	Energy use efficiency (g FW·kWh^-1^)	Energy use efficiency (g DW·kWh^-1^)
BFB-ETR 100	177 ± 11	8.07 ± 0.49	9.67 ± 0.41	3.63 ± 0.09	8.08	21.9 ± 1.4 a	1.00 ± 0.06 a
BFB-Φ_PSII_ 0.68	157 ± 13	7.54 ± 0.60	9.09 ± 0.19	3.63 ± 0.15	8.73	18.0 ± 1.5 b	0.86 ± 0.07 ab
ML-ETR 100	180 ± 12	8.31 ± 0.57	10.17 ± 0.46	4.00 ± 0.09	9.40	19.1 ± 1.3 ab	0.88 ± 0.06 ab
ML-Φ_PSII_ 0.68	179 ± 12	8.38 ± 0.42	9.79 ± 0.41	3.87 ± 0.16	9.86	18.2 ± 1.2 ab	0.85 ± 0.04 ab
PPFD 350	171 ± 6	7.70 ± 0.24	9.68 ± 0.35	3.71 ± 0.15	9.67	17.7 ± 0.6 b	0.80 ± 0.02 b
*P*-values	0.23	0.38	0.37	0.079		0.006	0.011

Parameters include shoot fresh weight, shoot dry weight, leaf chlorophyll and anthocyanin content, total energy use, and energy use efficiency.

Values are means ± standard errors (n = 5). Different letters within each column indicate significant differences at α = 0.05, according to Tukey’s HSD test.

## 4 Discussion

The MLR model developed in this study provided physiologically interpretable relationships between Φ_PSII_ and environmental variables. The direction and magnitude of the regression coefficients were consistent with established physiological responses reported in previous studies, supporting the biological plausibility of the model structure. Although prediction errors remained, the model captured the primary variability in Φ_PSII_ and achieved predictive performance adequate for dynamic light regulation. Thus, the model balanced interpretability and predictability in a manner suitable for real-time biofeedback control applications.

Light intensity emerged as the dominant predictor of Φ_PSII_ across analyses (**Figure 2**, **Table 1**), consistent with its central role in regulating photosynthesis. While increasing irradiance enhances photosynthetic rate up to light saturation point, photosynthetic efficiency typically declines under higher light due to increased non-photochemical dissipation (Wimalasekera, 2019). This general physiological response was reflected in the model, where light intensity showed a negative relationship with Φ_PSII_ (**Table 2**) and remained among the most influential variables in the final model (**Figure 2**).

Among light-related variables, ePPFD showed slightly stronger bivariate relationship with Φ_PSII_ than PPFD (**Table 1**). In the final model, ePPFD was retained whereas PPFD was excluded, indicating that accounting for far-red spectral information improved predictive performance. Far-red photons preferentially excite photosystem I (PSI) over PSII, since the absorption peak of the PSI reaction center is 700 nm (Zhen et al., 2021). Although Φ_PSII_ quantifies PSII photochemical efficiency, PSII performance is coupled to downstream electron transport through PSI. Consequently, ePPFD, which includes far-red wavelengths beyond the conventional PAR range, was a more physiologically relevant predictor of Φ_PSII_ than PPFD and contributed to improved model performance.

Additionally, 15-min averaged light intensity showed higher explanatory power than instantaneous light intensity for both ePPFD and PPFD (**Table 1**), with ePPFD15 also exhibiting greater variable importance than ePPFDi in the final model (**Figure 2**). While light absorption and initial electron transfer occur on picosecond timescales (Baikie et al., 2023), overall photochemical efficiency is governed by relatively slower processes, such as NPQ dynamics and stomatal regulation (Lawson and Blatt, 2014; Matuszyńska et al., 2016). Consequently, short-term light history provides a critical temporal context for predicting Φ_PSII_. Nevertheless, MLR models incorporating both 15-min averaged and instantaneous light intensities outperformed those using averaged light alone, indicating that immediate responses to light fluctuations still contribute significantly to Φ_PSII_ variability.

CO_2_ concentration is a major regulator of photosynthesis, particularly in C_3_ plants, such as lettuce, following light intensity (Johnson et al., 2021). Elevated CO_2_ enhances photosynthetic efficiency by increasing the carboxylation efficiency of ribulose-1,5-bisphosphate carboxylase/oxygenase (Rubisco) and suppressing photorespiration (Villagran et al., 2025). Although CO_2_ was not actively enriched in the experimental greenhouse, natural fluctuations in CO_2_ concentration likely contributed to the positive CO_2_ coefficient observed in the model (**Table 2**).

Along with light intensity and CO_2_ concentration, VPD is a key driving force of photosynthesis because it regulates stomatal conductance and transpiration (Lauwers et al., 2025). Excessively high VPD reduces stomatal conductance, whereas very low VPD limits transpiration and associated CO_2_ uptake (Amitrano et al., 2021). VPD values above approximately 1 kPa are generally considered stressful for many crops. In this study, however, most VPD values were within 0–1 kPa (**Figure 1**). Within this range, increasing VPD may have been associated with enhanced transpiration and stomatal conductance, resulting in a positive relationship between VPD and Φ_PSII_ in the model (**Table 2**).

Temperature had the lowest relative importance among the environmental variables included in the model (**Figure 2**) but exhibited a significant negative coefficient in the prediction of Φ_PSII_ (**Table 2**). Photochemical efficiency is generally constrained outside an optimal temperature range (Neri et al., 2024). In lettuce, Φ_PSII_ has been reported to increase from 15 °C to 23 °C and to decline slightly as temperature increases further to 30 °C under prolonged exposure (Zhou et al., 2019). In the present study, the temperature range was largely limited from optimal to moderately high conditions (**Figure 1**). Consequently, the model captured only the declining portion of the temperature–Φ_PSII_ response, resulting in a negative coefficient rather than a quadratic relationship (**Table 2**). This VPD and temperature result underscores that model structure and interpretation depend on the specific environmental conditions represented in the training data.

However, environmental variables alone did not fully account for the diurnal pattern of Φ_PSII_ characterized by an afternoon decline in Φ_PSII_ (**Figure 4**). Incorporating a binary Time-of-Day variable improved predictive performance, suggesting that internal physiological processes or cumulative constraints influenced photochemical activity beyond environmental conditions. One possible explanation is carbohydrate feedback inhibition, where starch or sugar accumulation during the day can downregulate PSII efficiency (Adams III et al., 2014). In addition, the slow relaxation of photoinhibitory NPQ (qI) may lead to a gradual reduction in Φ_PSII_ that requires several hours of recovery (Kono and Terashima, 2014). Such diurnal patterns may also reflect regulation by the endogenous circadian clock, which can modulate photosynthetic efficiency independently of external environmental cues as the photoperiod progresses (García‐Plazaola et al., 2017; Yarkhunova et al., 2018).

However, Time-of-Day functions as a proxy variable rather than representing a direct physiological mechanism. In preliminary analyses, cumulated DLI and eDLI variable was tested as an alternative predictor to represent cumulative light exposure. But DLI and eDLI showed very low variable importance in the preliminary model without improving model performance compared with the model including Time-of-Day. Although Time-of-Day improved predictive accuracy in this study, models relying on absolute time may be less transferable to other greenhouse systems operating under different photoperiod regimes, such as extended photoperiods or continuous lighting. Therefore, developing predictive models that incorporate variables relevant to those lighting regimes may be necessary for broader application.

In the validation experiment, both sensor- and ML-based ETR logics precisely maintained the target ETR of 100 μmol·m^-2^·s^-1^ with relatively stable PPFD adjustments (**Figures 7**, **8**). In contrast, Φ_PSII_-based logics exhibited substantially greater PPFD fluctuations in both sensor- and ML-based control (**Figures 7B, D**). A similar distinction between ETR- and Φ_PSII_-based control was previously observed under controlled growth chamber conditions (Nam et al., 2025), suggesting that this behavior reflects inherent characteristics of the control logic rather than environmental variability in the greenhouse. This difference arises from the structural relationship between ETR and PPFD. Because ETR is calculated as a function of Φ_PSII_ and incident PPFD (Maxwell and Johnson, 2000), leading to buffered light adjustments. In contrast, although Φ_PSII_ is influenced by irradiance, PPFD is not explicitly embedded in its calculation. Consequently, transient changes in photochemical efficiency translate more directly into dynamic regulation of LED lighting.

Importantly, these differences do not indicate that one approach is inherently superior, but rather that they pursue different physiological priorities. ETR-based control effectively compensates for changes in photochemical efficiency in order to maintain a specific photosynthetic activity (van Iersel et al., 2016b). In contrast, Φ_PSII_-based control stabilizes photochemical efficiency by lowering PPFD when Φ_PSII_ declines to alleviate light-induced stress, or by increasing PPFD when efficiency improves, so that light can be used more effectively. Thus, the selection of control logic, either ETR or Φ_PSII_, depends on whether the production goal prioritizes consistent photosynthetic activity for growth or resilient photochemical efficiency under stress conditions.

Meanwhile, Φ_PSII_ under the constant PPFD treatment exhibited relatively greater temporal variability than in all biofeedback treatments (**Figure 9E**). This indicates that maintaining a fixed light intensity does not prevent fluctuations in photochemical efficiency driven by environmental variability and physiological acclimation processes (Nam and Ferrarezi, 2026). In contrast, biofeedback control dynamically adjusted PPFD in response to real-time plant status, thereby stabilizing photochemical activity and buffering physiological variability that cannot be mitigated by constant light supply alone.

The initial targets of PPFD 350 μmol·m^-2^·s^-1^, ETR 100 μmol·m^-2^·s^-1^, and Φ_PSII_ 0.68 was selected from preliminary trials because they produced similar DLI values, thereby enabling comparison of the different control logic under approximately equivalent light input. However, at the beginning of each photoperiod in the validation experiment, the BFB-Φ_PSII_ 0.68 treatment had a Φ_PSII_ value of approximately 0.60 at a PPFD of 350 μmol·m^-2^·s^-1^ (**Figure 9B**). PPFD was therefore reduced to achieve the target Φ_PSII_, resulting in a lower DLI than in other treatments (**Figure 3**). Φ_PSII_-based control directly regulates light according to photochemical efficiency and is therefore more sensitive to deviations between target and measured Φ_PSII_ than ETR-based control. Consequently, lower Φ_PSII_ values at the beginning of the photoperiod resulted in sustained reductions in PPFD and lower DLI in the sensor-based Φ_PSII_ treatment. Additionally, the greater day-to-day DLI variability in the sensor-based Φ_PSII_ treatment (**Figure 3**) likely reflects plant- or leaf-specific responses, as measurements were obtained from a single plant with the measurement spot repositioned daily. In previous work, sensor-based Φ_PSII_ control distinguished crop-specific photochemical characteristics between lettuce and cucumber more sensitively than ETR control, resulting in differential light regulation between species (Nam et al., 2025). In contrast, the ML-based Φ_PSII_ treatment maintained PPFD within the intended range and resulted in DLI values comparable to other treatments. The ML-based Φ_PSII_ treatment relied on a model trained on multiple plants, capturing more general physiological patterns and yielding more consistent daily light input. These differences highlight that sensor- and ML-based approaches differ in their sensitivity to plant- and leaf-level variability.

In the ML-based and constant PPFD treatments, Φ_PSII_ was predicted to decline gradually during the morning hours (**Figures 9C, E**), whereas sensor-based treatments showed relatively stable Φ_PSII_ (**Figures 9A, B**). In the training dataset, CO_2_ declined while light intensity increased toward midday. The combined diurnal shifts in these variables led to a gradual morning decrease in predicted Φ_PSII_. In the validation experiment, however, light intensity was maintained within a narrower range at a moderate level of approximately 350 μmol·m^-2^·s^-1^ using supplemental lighting, whereas CO_2_ and other microclimate variables retained similar diurnal patterns. Under these stabilized light conditions, the predicted morning decline in Φ_PSII_ was not observed in the actual CF measurements using the fluorometers.

The influence of CO_2_ on photosynthesis depends on irradiance level. At higher light, photosynthesis becomes more responsive to CO_2_ concentration, whereas under low and moderate irradiance, light availability constrains the response to changes in CO_2_ (Körner et al., 2009; Zhao et al., 2021). Thus, decreasing CO_2_ in the morning likely had a different effect on Φ_PSII_ during the validation experiment compared with the high-variable light conditions of the training period. Future model development may benefit from incorporating interaction terms (PPFD × CO_2_) to account for the irradiance-dependent effects of CO_2_ on Φ_PSII_, although preliminary evaluation of the interaction term in the present dataset resulted in only marginal improvements in predictive performance.

Importantly, light intensity was both a key explanatory variable in model development and the primary control variable in the biofeedback system. Capturing Φ_PSII_ responses across a broad light range was necessary during training; however, supplemental lighting stabilized light intensity during the validation experiment. This inherent difference in light distribution between model development and validation likely contributed to the observed differences in Φ_PSII_ diurnal patterns. However, a model developed under a narrower light range would likely be restricted to a limited set of ETR and Φ_PSII_ targets. In contrast, models trained across a broader range of light intensities may allow more flexible selection of ETR and Φ_PSII_ targets during implementation.

Overall, sensor-based control reflects real-time plant responses under the actual experimental environment and can more directly capture plant-specific differences in photochemical characteristics. However, because light regulation is based on measurements from a portion of a single leaf, it may be more sensitive to variability among plants and leaves. In contrast, ML-based control represents generalized physiological patterns derived from multiple plants during model development, resulting in more consistent light regulation. Nevertheless, when the environmental conditions during application differ from those represented in the training dataset, predicted Φ_PSII_ may deviate from real-time measurements. For practical implementation, training datasets collected under environmental conditions similar to the intended application may improve model performance and robustness.

Despite differences in light regulation patterns, no significant differences in plant growth were observed among treatments (**Table 5**). This suggests that cumulative light supply was sufficient in all treatments to support comparable carbon assimilation and biomass accumulation (**Figure 3**). In contrast, only the sensor-based ETR control treatment reduced total LED lighting energy use and improved energy use efficiency compared with the constant PPFD treatment (**Table 5**). The sensor-based ETR control stabilized photosynthetic activity according to real-time plant physiological status while reducing unnecessary light input. In comparison, ML-based control may not fully capture complex plant responses under experimental conditions. More complex predictive approaches may provide modest improvements in prediction accuracy of Φ_PSII_, however, their increased complexity may limit interpretability and implementation in real-time lighting control systems.

Additionally, both sensor- and ML-based Φ_PSII_ control responded sensitively to small fluctuations in CF measurements compared to ETR control, which may not consistently translate into improved energy use efficiency. Although sensor-based ETR control may have limitations related to fluorometer cost and the limited representativeness of leaf-level measurements, recent developments in chlorophyll fluorescence instrumentation may help overcome these constraints. For example, newer systems capable of simultaneously measuring multiple leaves, such as multi-head PAM fluorometers (e.g., MICRO-PAM, Heinz Walz GmbH), can improve canopy representativeness while reducing the cost barrier for larger-scale deployment. Given that sensor-based ETR control improved LED lighting energy use efficiency in this study, further advances in CF sensing technologies may enhance the practicality of implementing plant-driven lighting regulation in greenhouse production.

## 5 Conclusion

This study provides a proof-of-concept demonstration of integrating an MLR model into a real-time biofeedback LED lighting control. Sensor-based control directly reflected plant physiological status under specific experimental conditions, whereas ML-based control regulated lighting based on generalized response patterns derived from training data. Compared with constant PPFD, both sensor- and ML-based approaches stabilized photochemical efficiency across the photoperiod. Although biomass accumulation did not differ among treatments, sensor-based ETR control achieved the highest energy use efficiency, indicating that real-time plant-driven regulation can reduce unnecessary light input. Future research may further evaluate model robustness across diverse crops and environmental conditions and explore advanced modeling approaches to enhance predictive reliability in commercial greenhouse applications.

## Data Availability

The raw data supporting the conclusions of this article will be made available by the authors, without undue reservation.
