# Supplementary material for: Chlorophyll fluorescence-based control of greenhouse supplemental lighting improves energy use efficiency in lettuce
Source: Front Plant Sci. 2026 Jul 13;17:1854406. doi: 10.3389/fpls.2026.1854406 (PMC13402182; doi:10.3389/fpls.2026.1854406)
Supplement: Supplementary file 1 [file SupplementaryFile1.docx]

Supplementary Material

# Supplementary Figure


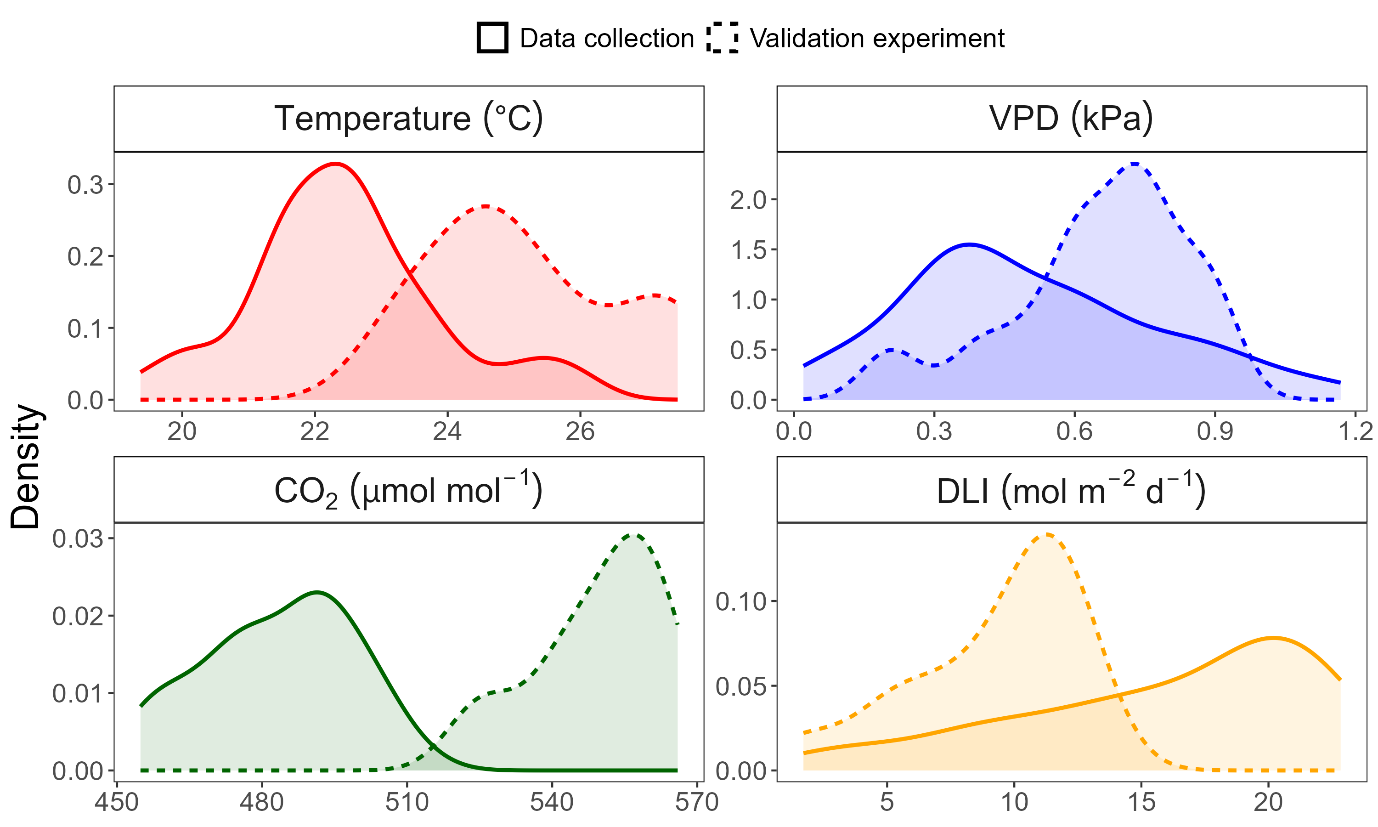


**Supplementary Figure 1.** Distribution of environmental conditions during the data collection period and validation experiment. Density plots represent the distributions of air temperature, vapor pressure deficit (VPD), CO_2_ concentration, and daily light integral (DLI). Solid and dashed lines indicate the data collection and validation periods, respectively.


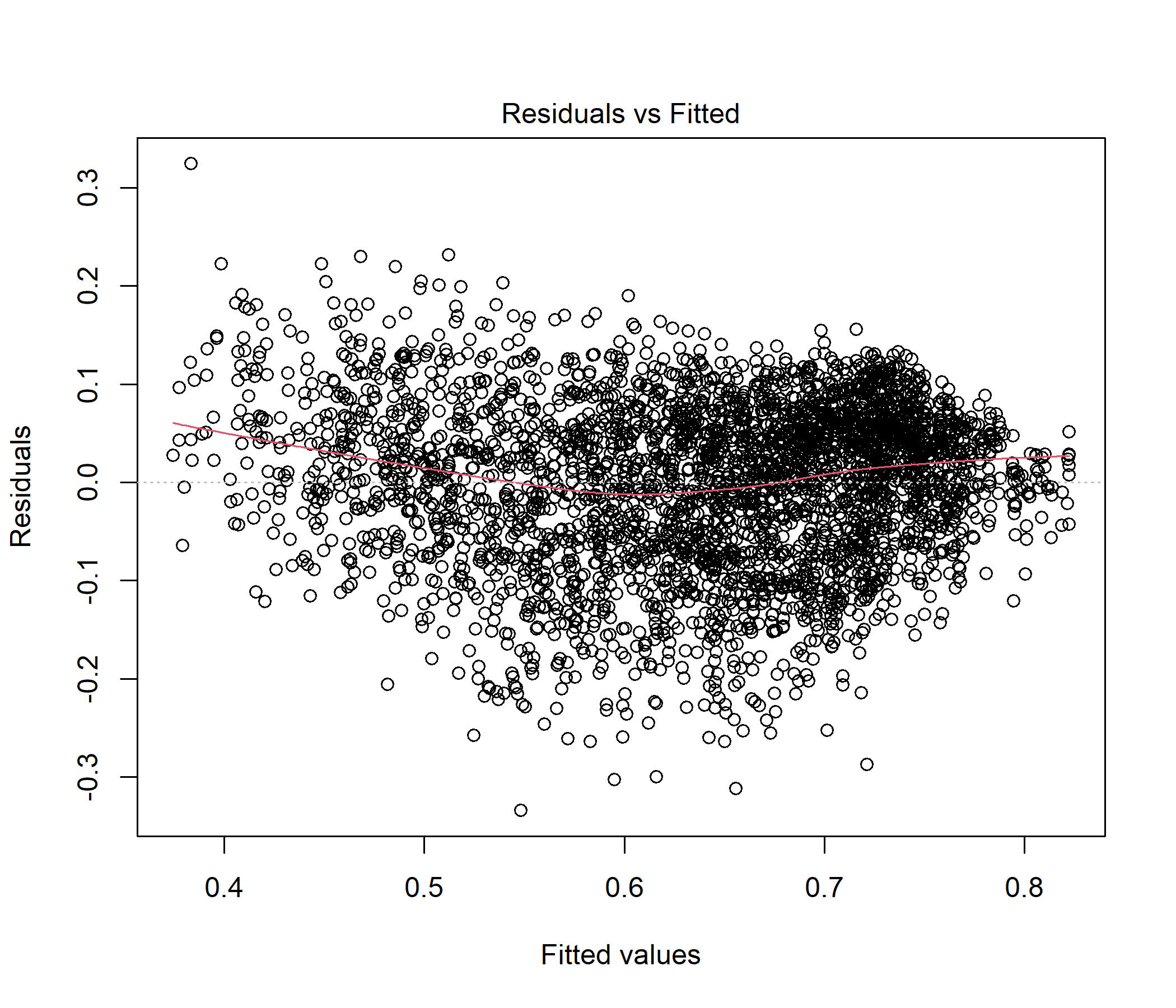


**Supplementary Figure 2.** Residuals-versus-fitted plot for the final multiple linear regression (MLR) model predicting Φ_PSII_.


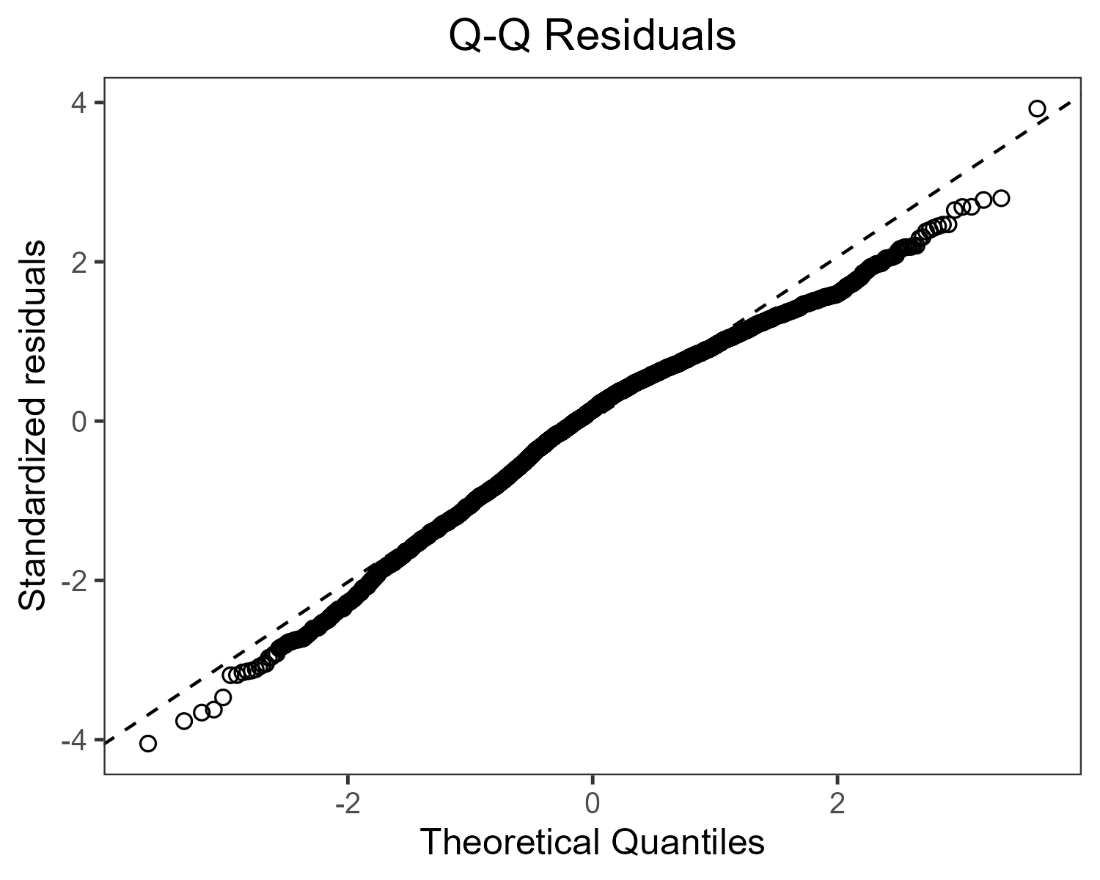


**Supplementary Figure 3.** Normal Q-Q plot of residuals from the final multiple linear regression (MLR) model predicting Φ_PSII_.

**Supplementary Table 1.** Variance inflation factors (VIF) for predictor variables included in the final multiple linear regression model.

| Predictors | VIF |
| --- | --- |
| ePPFD15 | 4.108 |
| ePPFDi | 3.705 |
| Temp | 1.569 |
| VPD | 1.632 |
| CO_2_ | 1.909 |
| Time-of-day^1^ | 1.786 |
